# Comparative Analysis of Physicochemical Properties and Volatile Profile of Eight Varieties of Green Plums in Sichuan and Yunnan

**DOI:** 10.3390/foods15061057

**Published:** 2026-03-17

**Authors:** Mengsheng Deng, Xingyong Zhang, Shuang Li, Wenao Sun, Huina Li, Chuan Song, Rui Huang, Zonghua Ao, Zhiping Fan, Dong Li

**Affiliations:** 1 School of Food and Liquor Engineering, Sichuan University of Science & Engineering, Yibin 644000, China; dsqc87@suse.edu.cn (M.D.); z17770848745@163.com (X.Z.); lishuang@suse.edu.cn (S.L.); swa12222@163.com (W.S.); lihuina1228@163.com (H.L.); 2 Luzhou Laojiao Co., Ltd., Luzhou 646000, China; songchuan@lzlj.com (C.S.); huangrui1@lzlj.com (R.H.); aozh@lzlj.com (Z.A.); 3 Yibin Center of Inspection and Testing, Yibin 644000, China; 18181679815@163.com

**Keywords:** green plum cultivars, organic acid, principal component analysis, volatile compound

## Abstract

The physicochemical properties and volatile composition of fruits are critical determinants of fruit quality and processing performance. This study evaluated major green plum cultivars from Sichuan and Yunnan Provinces by analyzing fruit morphology, nutritional composition, bioactive compounds, and volatile profiles. Multivariate statistical analyses, including orthogonal partial least squares discriminant analysis (OPLS-DA), principal component analysis (PCA), and cluster analysis (CA), were applied to comprehensively assess cultivar-dependent quality differences. EH exhibited the highest total acid and glucose contents, whereas MD showed superior soluble solids, total sugars, solid–acid ratio, and several organic acids and sugars. Yunnan cultivars generally showed higher flavonoid contents and stronger antioxidant activities than Sichuan cultivars. Citric acid was the predominant organic acid. A total of 97 volatile compounds were identified. Ten volatile compounds were detected in all eight varieties, including butyl acetate, hexyl acetate, and butyl butyrate. EH and MD released the higher volatile, and PCA-based comprehensive evaluation ranked the cultivars as follows: EH, MD, EY, YZ, PX, EZ, DY and DN. Therefore, EH and MD exhibited superior overall quality in physicochemical properties and volatile composition. These findings provide a theoretical basis for evaluating green plum quality and their rational utilization in production and processing.

## 1. Introduction

The green plum (*Prunus mume* Sieb. et Zucc.) is the fruit of green plum trees belonging to the Rosaceae family and is widely distributed across East and Southeast Asia, including China, Japan, and Korea [1]. The species is native to southern China and has been cultivated for more than 3000 years [2,3]. Green plums are rich in organic acids, sugars, vitamins, and essential trace elements, as well as bioactive compounds such as phenolic acids and flavonoids [4]. Owing to their characteristic high acidity and the presence of cyanogenic glycosides such as amygdalin and prunasin, fresh green plums exhibit an undesirable flavor profile and are not suitable for direct consumption [5]. Moreover, as climacteric fruits, green plums have a short postharvest shelf life, which further limits their fresh marketability. Consequently, green plums are predominantly processed into value-added products, including dried plums (e.g., medicinal ume), fruit wines, and fruit vinegars [6]. However, substantial quality differences have been observed among products derived from different green plum cultivars. Consequently, a comprehensive evaluation of the quality attributes of different green plum varieties is essential to determine their suitability for specific processed products.

China possesses abundant genetic resources of green plum, with more than 205 cultivars reported, providing a solid raw material basis for the development of diversified green plum products [7]. Substantial differences have been observed among cultivars in terms of total sugar, total acid, and vitamin contents. For example, the Fujian Qingzhu plum is characterized by large fruit size, a high sugar–acid ratio, and moderate Vitamin C content, whereas the Longyan plum exhibits only 35.40% and 42.11% of the sugar–acid ratio and Vitamin C content of Qingzhu plum, respectively. Nevertheless, its relatively high total phenolic and flavonoid contents provide a favorable biochemical basis for functional food processing [8]. Similarly, the Yingsu plum, owing to its high levels of total phenolics and flavonoids, has become an important raw material for deep processing into functional foods, cosmetics, and related products [5]. Previous studies have demonstrated that green plums with distinct nutritional profiles are suitable for different processing applications. Cultivars with high sugar–acid ratios and appropriate levels of vitamins and minerals are more suitable for the production of dried and candied plums, whereas cultivars rich in flavonoids are preferable for beverage processing. In addition, the high acidity and abundant amino acids of certain green plum cultivars make them ideal raw materials for seasonings and health supplements [9]. Based on PCA analysis, Jiang et al. [8] conducted a comprehensive ranking of green plum cultivars using sugar–acid ratio, total phenolic content, and flavonoid content as key indicators to identify suitable raw materials for product processing. In addition to sugar–acid ratio and phenolic content, fruit nutritional value and processing performance are strongly influenced by the composition and concentration of volatile aroma compounds [10,11,12,13]. However, existing studies on green plum aroma characteristics have focused on a limited number of cultivars, such as Nangao plum and Baifen plum, and are largely restricted to representative cultivars from major production regions, including Baifen plum from Fujian and Qingzhu plum from Guangdong. Liu et al. [14] identified 77 volatile compounds and found that ethyl butyrate, β-laurolene, 3-methylbutyl acetate, benzaldehyde, and nonanal were the key characteristic aromas in green plums. Further study suggested that in Yingsu plum and Changnong plum, aldehydes and ketones predominated at the immature and commercial maturity stages, whereas esters and alcohols became dominant at full maturity [15]. Consistently, the fully ripe Yingsu plum contained higher levels of volatile compounds than Changnong plum, which was closely associated with the predominance of esters and alcohols in Yingsu plum, while Changnong plum was mainly characterized by aldehydes and terpenes [16]. Taken together, physicochemical properties and volatile components are major determinants of quality differences among green plum cultivars.

The growth area significantly influences green plum quality, such as sugar–acid ratio and aroma profiles [8,15,16].

The southwest region of China, primarily encompassing Sichuan and Yunnan Provinces, is one of the country’s major green plum production areas. This region is characterized by a subtropical monsoon climate with warm and humid conditions and mild winters. Its complex mountainous terrain, together with well-drained and slightly acidic soils, provides a highly suitable ecological environment for green plum cultivation [17]. The southwest region harbors abundant green plum cultivar resources, including well-known varieties such as Nangao plum, Zhaoshui plum, Yingsu plum, and Xing plum. Commercial green plum production is mainly concentrated in counties such as Dayi, Mabian, and Pingwu in Sichuan Province, as well as Eryuan and Yulong in Yunnan Province. In Dayi County, Yingsu plum is the dominant cultivar and is characterized by large fruit size and high acidity [18]. Nangao plum, introduced into Pingwu County, is valued for its high yield, whereas the local Xing plum is distinguished by its unique flavor profile. In Mabian County, the primary cultivars include Nangao plum, Yingsu plum, and Dabai plum, accompanied by a wide diversity of wild germplasm resources. In Eryuan County of Yunnan Province, Zhaoshui plum is the predominant cultivar and is widely utilized for deep processing and plum wine production. Other cultivated varieties, such as Yan plum and Huang plum, also perform well. Previous studies have investigated the physicochemical properties and volatile components of a few varieties of green plum cultivars from the southwest region; for instance, they analyzed the fruit quality characteristics of cultivar D-1 and the volatile profiles of Dazhou plum [15,16]. However, no studies have reported on the physicochemical properties and volatile components of the main green plum cultivars in Sichuan and Yunnan Provinces, as cultivar and ecological conditions are important factors influencing fruit quality.

Therefore, a systematic investigation of the fruit quality characteristics of green plums from Sichuan and Yunnan is of substantial scientific and practical significance. This study selected fruits from eight green plum cultivars in Southwest China to identify high-quality varieties. Key attributes such as appearance, total phenolic content, and antioxidant capacity were analyzed, alongside the volatile flavor profiles. Multivariate statistical methods (OPLS-DA and PCA) were used for integrated evaluation and cultivar discrimination. Composite quality scores were then calculated, and cultivar-specific recommendations for processing and utilization were made.

## 2. Materials and Methods

### 2.1. Fruit Materials

Eight major green plum cultivars, including Yulong Zhaoshui plum, Eryuan Yan plum, Eryuan Huang plum, Eryuan Zhaoshui plum, Pingwu Xing plum, Mabian Dabai plum, Dayi Nangao plum, and Dayi Yingsu plum, were selected as experimental materials (Table 1). Green plums are cultivated in flat, well-drained soils with high organic matter content, deep soil layers, and optimal aeration. The selected cultivation sites receive sufficient sunlight and are shielded from prevailing winds. Fruits at approximately 80% commercial maturity, free from visible pests and diseases, and with uniform color and size were harvested between mid-May and late June 2024. For each cultivar, six trees were randomly chosen, and 20 fruits were collected per tree from four orientations (east, west, south, and north), yielding a total of 120 fruits per cultivar. After harvest, the necessary fruits were immediately analyzed by GC-MS, and the rest of the green plums were stored at − 80 °C until further analysis.

### 2.2. Reagents

Methanol (HPLC grade; Fisher Scientific, Waltham, MA, USA) and acetonitrile (HPLC grade; Honeywell Trading Co., Ltd., Shanghai, China) were used as chromatographic solvents. Fructose, sucrose, glucose, malic acid, oxalic acid, citric acid, succinic acid, and tartaric acid (all HPLC grade) were purchased from Yuanye Bio-Technology Co., Ltd. (Shanghai, China). Zinc acetate, potassium ferrocyanide, gallic acid, sodium hydroxide, sodium carbonate, sulfuric acid, and potassium dihydrogen phosphate (all of analytical grade) were obtained from Kelong Chemical Co., Ltd. (Chengdu, China). A total antioxidant capacity assay kit was purchased from Comin Biotechnology Co., Ltd. (Suzhou, China).

### 2.3. Test Method

#### 2.3.1. Determination of Fruit Appearance Traits

The appearance traits of mature fruits were measured on the day of harvest. Single-fruit weight was determined from fresh weight using an analytical balance with a precision of 0.01 g. Fruit transverse and longitudinal diameters were measured using a vernier caliper.

#### 2.3.2. Determination of Soluble Solids, Total Sugars, and Total Acid

The soluble solids content (SSC) was measured using a digital refractometer (RP-101, Atago, Tokyo, Japan) [19]. Total sugar content was determined using the phenol–sulfuric acid method [20]. Total acid (TA) (expressed as citric acid equivalents) was determined by acid–base titration with an indicator [21].

#### 2.3.3. Determination of Fruit Fructose, Glucose, and Sucrose

The contents of fructose, glucose, and sucrose were determined using high-performance liquid chromatography (HPLC) with an Agilent 1260 system (Agilent Technologies, Santa Clara, CA, USA) [22]. An amount of 1.0 g of green plums was homogenized in 20 mL of ultrapure water. Then, 1.25 mL of 1 mol L^−1^ zinc acetate solution and 1.25 mL of 0.25 mol L^−1^ potassium ferrocyanide solution were added. The mixture was subsequently subjected to ultrasonic treatment for 30 min. The solution was then diluted to 25 mL and filtered through a 0.22 μm membrane filter before being analyzed by HPLC. Mixed standard solutions of fructose, glucose, and sucrose were prepared at concentrations of 10 mg L^−1^, 25 mg L^−1^, 50 mg L^−1^, 100 mg L^−1^, 200 mg L^−1^, and 400 mg L^−1^, for the construction of the standard curve. Stability, precision, repeatability, detection limit, and quantification limit tests were then performed. Chromatographic separation was performed on a Hypersil GOLD Amino column (4.6 mm × 250 mm, 5 μm). The mobile phase consisted of ultrapure water (A) and acetonitrile (HPLC grade, B) with isocratic elution at a ratio of 20:80 (A:B). The column temperature was maintained at 40 °C, and the injection volume was 10 μL. All compounds were identified based on the retention time of standards, and their concentrations were calculated from the external calibration curves of standards.

#### 2.3.4. Determination of Organic Acid

The determination of organic acid content was performed using an Agilent 1260 high-performance liquid chromatography (HPLC) system (Agilent Technologies, USA) [23]. An amount of 1.0 g of freeze-dried green plum powder (completely freeze-dried using a freeze dryer) was added to 50 mL of ultrapure water, followed by homogenization and centrifugation at 6000 rpm for 15 min. The supernatant was filtered through a 0.45 μm membrane filter and subsequently analyzed by HPLC. A total of 100 mg of citric acid, tartaric acid, and malic acid was accurately weighed, and 50 mg of oxalic acid and succinic acid was dissolved in a 90% (*v*/*v*) methanol–water solution and diluted to a final volume of 10 mL. The resulting solutions were further diluted to 1, 5, 10, 20, 30, and 50 times using potassium dihydrogen phosphate solution to prepare gradient mixed standard solutions. A calibration curve was constructed based on the concentrations of the standard solutions and their corresponding peak areas. Stability, precision, repeatability, detection limit, and quantification limit tests were then performed. Chromatographic separation was performed on a Zorbax SB-Aq column (4.6 mm × 150 mm, 5 μm). The mobile phase consisted of methanol (A) and 0.02 mol L^−1^ KH_2_PO_4_ solution adjusted to pH 3.0 with phosphoric acid (B), using isocratic elution at a ratio of 2:98 (A: B) and a flow rate of 0.6 mL min^−1^. The column temperature was maintained at 30 °C, UV detection was performed at 210 nm, and the injection volume was 10 μL. All compounds were identified based on the retention time of standards, and their concentrations were calculated from the external calibration curves of standards.

#### 2.3.5. Determination of Total Phenolic, Total Flavonoid, and Vitamin C

Total phenolic content (TPC) was determined using the Folin–Ciocalteu method as described previously [24]. Briefly, 5 g of green plum sample was extracted with 30 mL of 60% (*v*/*v*) ethanol and ultrasonicated for 10 min. The extract was then diluted to 50 mL with 60% ethanol, mixed thoroughly, and filtered. An aliquot (1.0 mL) of the filtrate was combined with 2.5 mL of Folin–Ciocalteu reagent, followed by 2.5 mL of 15% (*w*/*v*) sodium carbonate solution. The mixture was adjusted to volume with distilled water, incubated at 40 °C for 60 min, and cooled for 20 min, and the absorbance was measured at 778 nm. The results were expressed as milligram equivalents of gallic acid per 100 g of fresh weight (mg GAE 100 g^−1^)

Total flavonoid content (TFC) was determined using a colorimetric method as described previously [25]. Briefly, 0.5 g of green plum sample was homogenized with 1.5 mL of 80% (*v*/*v*) methanol in an ice bath and ultrasonicated for 30 min. The homogenate was centrifuged at 8000 rpm for 15 min at 4 °C, and the supernatant was collected and stored at 4 °C in the dark. An aliquot (1 mL) of the extract was mixed with 0.175 mL of 10% (*w*/*v*) aluminum nitrate solution. After reacting for 6 min, 1.25 mL of 4% (*w*/*v*) NaOH solution was added, and the mixture was allowed to stand for 15 min, after which the absorbance was measured at 510 nm. The results were expressed as milligrams of catechin equivalent (CE) per 100 g of fresh weight (mg CE 100 g^−1^).

Vitamin C (VC) content was determined using the 2,6-dichlorophenolindophenol titration method [26]. Approximately 10 g of green plum sample was frozen in liquid nitrogen and ground into a fine powder, then homogenized with 2 mL of 2% (*w*/*v*) oxalic acid solution. The homogenate was diluted to 100 mL with 2% oxalic acid solution and allowed to stand for 10 min, followed by centrifugation at 8000 rpm for 20 min at 4 °C. An aliquot (10 mL) of the extract was titrated with standardized 2,6-dichlorophenolindophenol solution. The endpoint was defined as the appearance of a faint pink color that persisted for 15 s, and the titrant volume was recorded. The VC content was calculated based on the titration volume and expressed as milligrams per 100 g of fresh weight (mg 100 g^−1^).

#### 2.3.6. Determination of Antioxidant Capacity

The antioxidant capacity of green plum fruits was assessed using commercial assay kits based on the 2,2-diphenyl-1-picrylhydrazyl (DPPH) and 2,2′-azinobis-(3-ethylbenzothiazoline-6-sulfonic acid) (ABTS) radical scavenging methods, following the manufacturers’ instructions.

#### 2.3.7. Determination of Volatile Flavor Compounds

Volatile flavor compounds in green plum fruit were analyzed using headspace solid-phase microextraction coupled with gas chromatography–mass spectrometry (HS-SPME-GC-MS), following the method described previously [27]. Approximately 3 g fresh green plum was placed into a 20 mL headspace vial, to which 1.4 g of sodium chloride and 20 μL of 1 mL/L 2-octanol solution (in anhydrous ethanol) were added as an internal standard. The vial was equilibrated at 45 °C for 15 min, and volatile compounds were subsequently extracted for 30 min. After extraction, the SPME fiber was inserted into the GC injection port and thermally desorbed at 210 °C for 7 min. GC–MS analysis was performed using a TSQ 8000 gas chromatography–mass spectrometry system (Thermo Fisher Scientific, Waltham, MA, USA). Gas chromatographic separation was performed using an MD-WAX capillary column (60 m × 0.25 mm, 0.25 μm). High-purity helium was used as the carrier gas at a constant flow rate of 1 mL/min. The injector was maintained at 250 °C and operated in splitless mode. The oven temperature program was as follows: 40 °C (held for 2 min), increased to 120 °C at 5 °C min^−1^ (held for 2 min), and then increased to 220 °C at 7 °C min^−1^ (held for 5 min). The total run time was 39 min. Mass spectrometric detection was performed using electron impact (EI) ionization at 70 eV. The transfer line, ion source, and quadrupole temperatures were set at 220 °C, 250 °C, and 150 °C, respectively. Mass spectra were recorded over an *m*/*z* range of 35–500 with a scan rate of 3.6 scans s^−1^.

Qualitative and quantitative analysis. Volatile compounds were identified by matching their mass spectra with entries in the NIST mass spectral library. Identification was further confirmed using retention time, spectral matching score, and retention index (RI). Quantification was performed using the internal standard method. The concentration of each volatile compound was calculated as follows: content of volatile compound = (peak area of the volatile compound × concentration of the internal standard)/peak area of the internal standard. For each compound, 2-octanol was used as the internal standard, and semi-quantitative analysis was conducted based on relative peak areas.

### 2.4. Data Processing and Statistical Analysis

Data were processed using Microsoft Excel 2024. OPLS-DA was performed with SIMCA 14.1, while PCA and one-way analysis of variance (ANOVA) were conducted using SPSS 27.0. All figures were generated using Origin 2024. Statistical significance was defined as *p* < 0.05, and highly significant differences were defined as *p* < 0.01. Unless otherwise stated, all measurements were carried out independently in triplicate (three biological replicates).

## 3. Results

### 3.1. Analysis of Fruit Appearance Traits

The appearance traits of fruits from eight green plum cultivars were evaluated. As shown in Table 2, significant differences (*p* < 0.05) were observed among cultivars in single-fruit weight, transverse diameter, and longitudinal diameter. Single-fruit weight ranged from 11.48 to 19.78 g, with PX exhibiting the highest value (19.78 g), followed by EZ, YZ, and EH, while DY had the lowest value (11.18 g). Fruit shape analysis indicated that EY and EZ fruits were nearly spherical, whereas the other cultivars were oblong. The highest fruit shape index values were observed in MD, DN, and DY.

### 3.2. Analysis of Soluble Solids, Total Sugars, and Total Acids

As shown in Table 3, the SSC of the eight green plum cultivars ranged from 6.03 to 10.81 °Brix. MD exhibited the highest SSC, which was significantly higher than those of the other cultivars (*p* < 0.05). No significant difference in SSC was observed between DN and DY (*p* > 0.05), with values of 6.03 and 6.10 °Brix, respectively. Total sugar content varied from 2.04 to 3.70%, with MD showing the highest value (3.70%), significantly exceeding that of the other cultivars (*p* < 0.05). EH followed with 3.36%, whereas PX had the lowest total sugar content (2.04%). Green plum is characterized by high acidity; TA ranged from 3.41 to 6.40%, with EH exhibiting the highest TA (6.40%), significantly higher than those of other cultivars (*p* < 0.05). No significant difference in TA was observed between YZ and EZ (*p* > 0.05), with values of 4.85% and 4.95%, respectively. The solid–acid ratio ranged from 1.55 to 1.80, with MD showing the highest value and DY the lowest. No significant differences in solid–acid ratio were found among YZ, EY, MD, and DN (*p* > 0.05). The sugar–acid ratio ranged from 0.37 to 0.76, with DN showing the highest value and PX the lowest. These results indicate that green plum is a high-acid, low-sugar fruit, rendering it generally unsuitable for fresh consumption and more appropriate for being processed into products.

### 3.3. Analysis of Fructose, Glucose, and Sucrose Contents in Fruit

Soluble sugars, mainly including fructose, glucose, and sucrose, affect the sweetness of green plum and the processing property of products. Fructose, glucose, and sucrose are the predominant sugars in green plum fruits [8]. As shown in Table 4, the concentrations of fructose, glucose, and sucrose varied significantly among cultivars, ranging from 0.36 to 2.17, 0.36 to 2.32, and 0 to 7.69 mg g^−1^, respectively. Among the eight cultivars, MD exhibited significantly higher fructose and sucrose contents than the other cultivars, with values of 2.17 and 7.69 mg g^−1^, respectively. EH had the highest glucose content (2.32 mg g^−1^), which was significantly higher than that of the other cultivars. Additionally, MD and EH had the highest total soluble sugar contents, at 11.96 and 6.79 mg g^−1^, respectively, consistent with their high total sugar levels. In contrast, DY showed the lowest total soluble sugar content (0.72 mg g^−1^).

### 3.4. Analysis of Organic Acids in Fruits

Organic acids are widely distributed in fruits and vegetables and play a crucial role in determining fruit taste perception and overall flavor profile [16]. The analysis of organic acids in different green plum cultivars (Table 5) indicated that total organic acid contents in MD, DN, YZ, and EH were not significantly different. Citric acid was the predominant organic acid, ranging from 275.47 to 462.22 mg g^−1^, followed by malic acid (1.53–44.03 mg g^−1^), succinic acid (0.79–24.50 mg g^−1^), and oxalic acid (0.57–3.52 mg g^−1^). Tartaric acid was the least abundant (0–1.61 mg g^−1^) and was not detected in EH, PX, or DY. YZ had the highest citric acid content (462.22 mg g^−1^), with no significant difference compared to EH. The highest malic acid contents were observed in MD, DY, and PX (44.03, 43.87, and 37.46 mg g^−1^, respectively), whereas YZ, EZ, and EY exhibited the lowest malic acid levels (5.67, 2.12, and 1.53 mg g^−1^, respectively). DN contained significantly higher succinic acid than the other cultivars (24.50 mg g^−1^). Across all cultivars, citric acid content was approximately 5–12 times higher than malic acid content, indicating that green plum is a citric-acid-dominant fruit.

### 3.5. Total Phenolic, Total Flavonoid, and Vitamin C Contents in Fruits

Total phenolics, total flavonoids, and VC are key bioactive components in green plum, directly influencing its processing value and product quality. As shown in Table 6, significant differences in these bioactive indicators were observed among the eight cultivars. TPC ranged from 82.37 to 409.40 mg GAE 100 g^−1^, with EY exhibiting the highest level (409.40 mg GAE 100 g^−1^), followed by PX (359.70 mg GAE 100 g^−1^), YZ (347.89 mg GAE 100 g^−1^), EZ (296.82 mg GAE 100 g^−1^), EH (247.12 mg GAE 100 g^−1^), and DY (157.61 mg GAE 100 g^−1^). MD and DN showed the lowest values, representing only 20.05% and 22.25% of the EY level, respectively. TFC ranged from 4.26 to 21.13 mg CE 100 g^−1^, with five cultivars (including EZ, EH, and EY) exceeding 10 mg CE 100 g^−1^; EZ had the highest content (21.13 mg CE 100 g^−1^). In contrast, PX, DN, and MD had relatively low flavonoid contents (4.26, 5.22, and 5.57 mg CE 100 g^−1^, respectively). VC content also varied significantly among cultivars. EY had the highest VC content (4.30 mg 100 g^−1^), whereas PX and DY had the lowest levels (0.98 and 0.82 mg 100 g^−1^, respectively). The remaining cultivars exhibited moderate VC contents.

### 3.6. Analysis of Fruit Antioxidant Capacity

**Figure 1:**
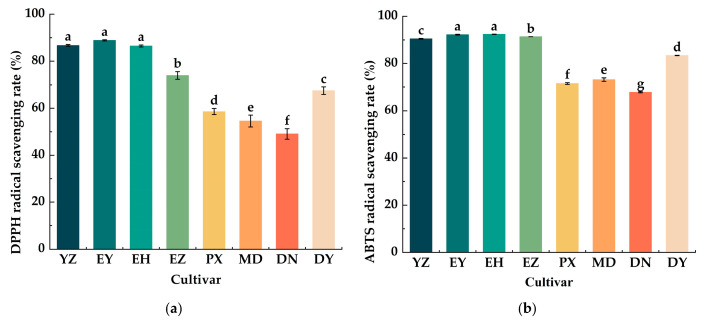
Antioxidant activities of eight green plum cultivars (**a**) DPPH radical scavenging activities of eight green plum cultivars. (**b**) ABTS radical scavenging activities of eight green plum cultivars. Different letters indicate significant differences among the eight cultivars (*p* < 0.05).

As shown in Figure 1, the ABTS radical scavenging capacities of all cultivars were higher than their corresponding DPPH values, and significant differences were observed among cultivars (*p* < 0.05). The DPPH assay revealed that EY, YZ, and EH exhibited the strongest radical scavenging activities, reaching 88.85%, 86.73%, and 86.52%, respectively, followed by EZ and DY. In contrast, PX, MD, and DN showed the lowest capacities, with values of 73.96%, 67.45%, 58.60%, 54.53%, and 49.09%, respectively. Notably, compared with the DPPH assay, the ABTS-scavenging capacities of EZ and MD increased, placing them between EH and YZ, and between DY and PX, respectively, whereas the ranking of the remaining cultivars remained unchanged. Total phenolics, flavonoids, and VC are key contributors to the antioxidant capacity of green plum. The high radical scavenging activities observed in EY, EH, and YZ were closely associated with their elevated levels of total phenolics, flavonoids, or VC. The correlation coefficient between total flavonoids and ABTS radical scavenging activity was 0.91, while that with DPPH radical scavenging activity was 0.79. Although EH and EZ exhibited moderate total phenolic contents, they showed the highest flavonoid levels among the eight cultivars, and a similar trend was observed in DY.

### 3.7. Analysis of Volatile Compounds in Fruits

Volatile compounds (VCs) in different green plum cultivars were profiled using gas chromatography–mass spectrometry (GC-MS). The aroma of green plum fruit is largely determined by the composition and relative abundance of volatile compounds, and significant differences in aroma profiles among cultivars arise from variations in their constituent profiles. As shown in Table 7, a total of 97 major volatile compounds were identified across the eight cultivars, comprising 31 esters (31.95%), 6 aldehydes (6.19%), 21 alcohols (21.65%), 11 ketones (11.34%), 8 acids (8.25%), and 20 other compounds, including alkenes and alkanes (20.62%). Ten volatile compounds were common to all cultivars, namely ethyl acetate, butyl acetate, pentyl acetate, hexyl acetate, butyl butyrate, ethyl octanoate, decanal, n-butanol, n-hexanol, and 2-octanone. EY exhibited the highest total volatile content (15,816.65 μg kg^−1^), followed by EH (11,883.46 μg kg^−1^) and MD (6993.52 μg kg^−1^). Esters were the most diverse class of volatiles across cultivars, and key esters such as ethyl acetate, butyl acetate, pentyl acetate, hexyl acetate, butyl butyrate, and ethyl octanoate contribute substantially to the characteristic fruity and floral aroma of green plum fruit. Consistent with this, EY had the highest ester content (14,677.96 μg kg^−1^), followed by EH (8552.44 μg kg^−1^) and MD (6766.12 μg kg^−1^).

**Figure 2:**
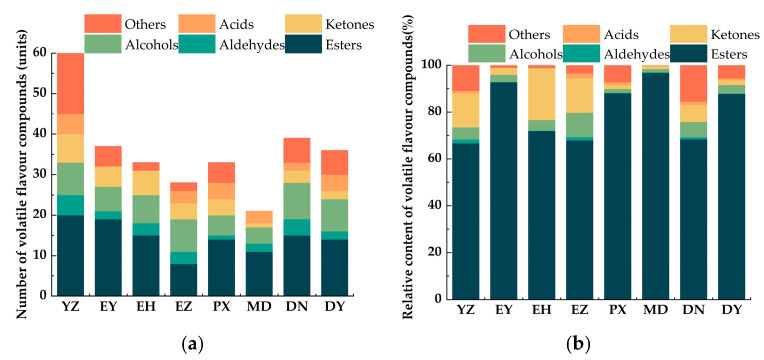
(**a**) Radar diagram of the number of volatile components in eight green plum cultivars. (**b**) Radar diagram of relative content of volatile components in eight green plum cultivars.

As shown in Figure 2, YZ exhibited the highest volatile compound diversity, with 60 compounds detected, accounting for 61.86% of the total identified compounds. In contrast, MD displayed the lowest diversity, with only 21 compounds detected. Across the eight cultivars, the volatile profiles were dominated by esters, alcohols, and ketones, with smaller proportions of aldehydes, acids, hydrocarbons, and phenolic compounds. Esters were the predominant contributors in all cultivars. In MD, esters accounted for the highest relative abundance (96.75%), followed by alcohols and ketones. Similarly, esters comprised 92.80% of the volatile profile in EY. YZ and EZ exhibited similar volatile compositions, with relatively high abundances of butyl acetate, hexyl acetate, and butyl butyrate, which may serve as characteristic aroma markers of green plum. Ketones and alcohols contributed substantially to the aroma differences among cultivars.

### 3.8. Orthogonal Partial Least Squares Discriminant Analysis (OPLS-DA) and Principal Component Analysis (PCA)

Although multiple quality attributes of different green plum cultivars were measured, the relative contribution of each attribute to cultivar differentiation remained unclear. Therefore, OPLS-DA and PCA were employed for comprehensive evaluation, aiming to identify the key indicators driving quality variation among cultivars.

**Figure 3:**
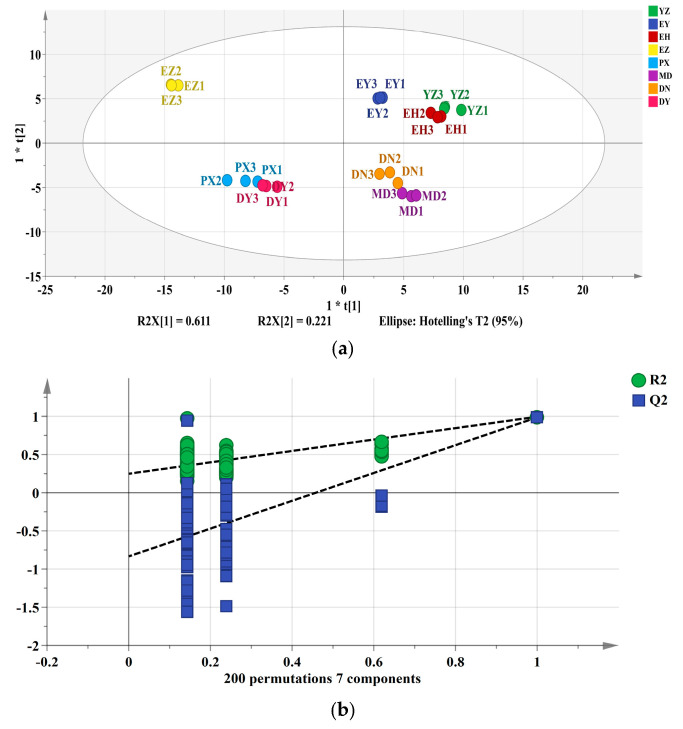

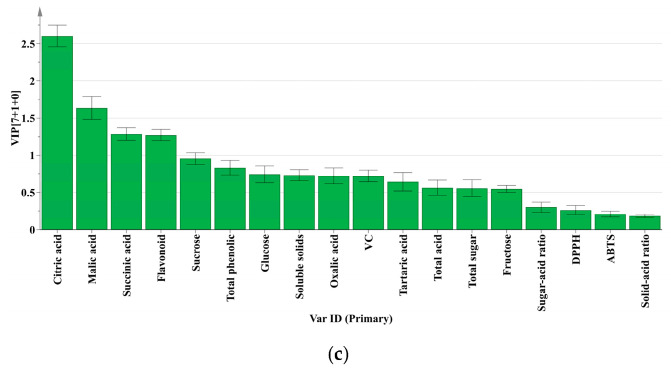
OPLS-DA analysis of eight green plum varieties. (**a**) Score plot of OPLS-DA based on multiple quality parameters of green plum fruits. (**b**) Permutation test of the OPLS-DA model. (**c**) Variable importance in projection (VIP) values of different parameters for green plum fruits.

PCA was conducted to reduce data dimensionality and comprehensively evaluate quality differences among cultivars. The results are presented in Figure 3. As shown in Figure 3a, all samples were located within the 95% Hotelling’s T^2^ confidence ellipse, indicating the absence of significant outliers and confirming the robustness of the dataset for multivariate analysis. To further clarify the metabolic determinants responsible for cultivar discrimination, OPLS-DA was performed. Figure 3b shows the permutation test for the OPLS-DA model. The Q^2^ intercept was below zero, excluding model overfitting and validating the robustness and predictive capability of the model. Key indicators contributing to cultivar differentiation were identified using OPLS-DA. Based on variable importance in projection (VIP > 1), citric acid (VIP = 2.60), malic acid (VIP = 1.63), succinic acid (VIP = 1.28), and flavonoids (VIP = 1.27) were the major contributors to inter-cultivar variation. Considering that citric acid, malic acid, and succinic acid are pivotal intermediates of the tricarboxylic acid (TCA) cycle, and in light of the clear separation observed in the PCA score plot, the findings suggest that cultivar-dependent quality variation is largely driven by differences in TCA cycle-associated organic acid metabolism and the differential accumulation of flavonoid secondary metabolites. Among these, citric acid exhibited the highest VIP value, suggesting it is the most critical marker for cultivar discrimination. PCA was performed using SPSS 27.0. As shown in Table S1, three principal component factors were extracted, and the cumulative contribution of eigenvalue large 1 reached 82.00%, indicating that these PCs comprehensively represent the overall quality characteristics of the eight cultivars. The principal component score matrix revealed significant differences in PC scores among cultivars. In the loading analysis, DPPH scavenging rate, glucose, and total phenolics exerted the greatest influence on PC1, PC2, and PC3, with loading values of 0.849, 0.842, and 0.571, respectively. Based on the factor loading matrix (Table S2), a composite quality model was constructed using the scores of the three principal components.

Y_1_ = 0.237 ∗ X1 + 0.336 ∗ X2 + 0.250 ∗ X3 + 0.318 ∗ X4 + 0.384 ∗ X5 + 0.372 ∗ X6 + 0.119 ∗ X7 − 0.385 ∗ X8 − 0.276 ∗ X9 + 0.132 ∗ X10 + 0.331 ∗ X11 + 127 ∗ X12

Y_2_ = 0.241 ∗ X1 + 0.266 ∗ X2 − 0.277 ∗ X3 + 0.315 ∗ X4 − 0.225 ∗ X5 − 0.255 ∗ X6 + 0.235 ∗ X7 − 0.072 ∗ X8 + 0.293 ∗ X9 + 0.428 ∗ X10 − 0.245 ∗ X11 + 0.436 ∗ X12

Y_3_ = − 532 ∗ X1 + 0.358 ∗ X2 + 0.516 ∗ X3 + 0.303 ∗ X4 − 0.045 ∗ X5 − 0.165 ∗ X6 − 0.374 ∗ X7 − 0.001 ∗ X8 + 0.140 ∗ X9 + 0.029 ∗ X10 − 0.199 ∗ X11 + 0.031 ∗ X12

A comprehensive quality evaluation model for green plum was established based on the eigenvalues of the three extracted principal components:

Y = 0.408 ∗ y_1_ + 0.310 ∗ y_2_ + 0.102 ∗ y_3_

The comprehensive ranking presented in Table 8 was derived from the composite scores, where higher values indicate superior quality. Among the eight cultivars, EH ranked first, and the overall quality hierarchy was as follows: EH, MD, EY, YZ, PX, EZ, DY, and DN. These results further confirm the superior quality of EH and MD, despite differences in their physicochemical profiles.

### 3.9. Cluster Analysis

**Figure 4:**
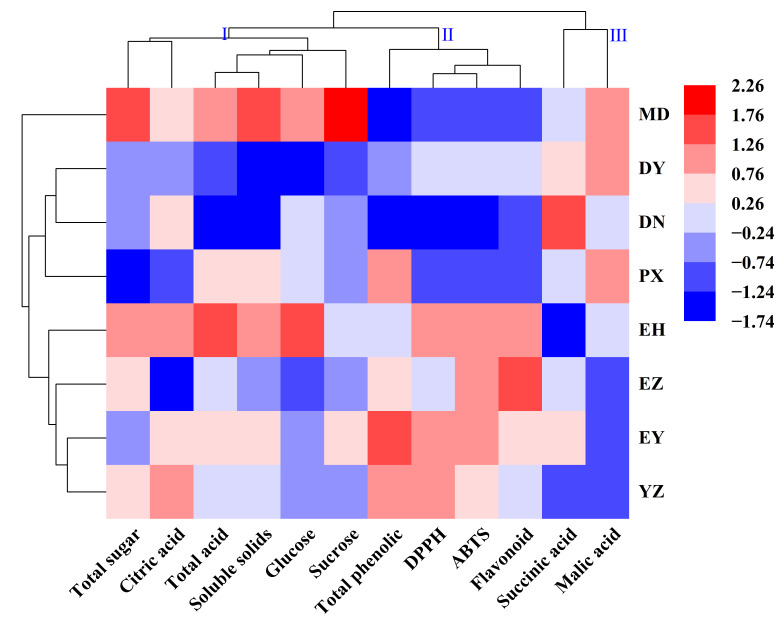
Cluster analysis of quality parameters of fruits from eight green plum cultivars.

Subsequently, CA was performed on the eight green plum cultivars (Figure 4). The physicochemical indicators used for clustering were categorized into three groups. Category I comprised six indicators, including total sugar, citric acid, TA, and SSC. Category II consisted of five indicators, such as total phenolics, DPPH radical scavenging capacity, and flavonoids. The remaining indicators, succinic acid and malic acid, were classified as Category III. Based on these three categories, the eight cultivars were divided into three clusters. The first cluster contained only MD, which was characterized by high levels of SSC, total sugar, and sucrose, suggesting its suitability for high-value product development, such as green plum wine and beverages. The second cluster included DY, DN, and PX, which exhibited relatively low levels of Category I and II substances (e.g., total sugar, TA, and antioxidant-related compounds) but higher contents of succinic acid and malic acid, resembling the Category III profile observed in MD. The third cluster comprised EH, EZ, EY, and YZ, which showed relatively lower succinic acid and malic acid levels, but more balanced Category I and II profiles. These cultivars also exhibited high TA, total phenolics, flavonoids, and antioxidant activities, indicating their potential for deep processing. Notably, discrepancies were observed between the CA and the comprehensive scoring results, which may be due to differences in the weighting of physicochemical parameters. Although MD and EH ranked closely in the comprehensive evaluation, they were not clustered together; nevertheless, both cultivars exhibited similarly high levels of Category I compounds.

## 4. Discussion

Although green plums are unsuitable for direct consumption due to their high acidity, their abundant sugars, organic acids, VC, and other bioactive compounds render them well aligned with human health needs. Consequently, green plums are commonly processed into products such as dried fruits and fruit wines. Therefore, a systematic evaluation of the physicochemical properties and aroma profiles of different green plum cultivars is essential. This need is particularly pressing in Southwest China, where green plum resources are abundant but comprehensive quality assessments remain limited compared with those in East and South China. In the present study, eight green plum cultivars were comprehensively evaluated for their sugar and organic acid composition (Table 4 and Table 5), antioxidant capacity (Figure 1), and volatile aroma compounds (Table 7). Among the cultivars, EH and MD exhibited superior overall quality, providing valuable references for the development and utilization of green plum resources. Regrettably, our study only compared the quality differences between green plum cultivars, with limited consideration of environmental factors such as light, temperature, and water; after all, YZ differs from EZ in total sugar, organic acid, etc.

Physicochemical characteristics reflect the intrinsic quality of green plum fruits and strongly influence their taste balance and processing performance. In the study, the SSC of the eight green plums ranged from 6.03 to 10.81 °Brix (Table 4), with the minimum value exceeding the lower bound reported by Liu et al. [28]. Green plums from Southwest China generally exhibit higher SSC than those from Jiangsu and Zhejiang, consistent with the findings of Chen et al. [16]. Among the cultivars tested, MD and EH showed significantly higher SSC than the others (Table 3), corresponding to their higher total sugar and TA. The results indicated that MD is a typical high-sucrose and high-fructose cultivar, exhibiting stronger sweetness and a relatively mild taste, making it suitable for processed products such as plum wine and beverages. EH contained the highest glucose concentration; its sweetness was moderate, but combined with high acidity, it contributed to a more intense flavor profile. Notably, differences in glucose and fructose contents among cultivars were not significant, a phenomenon also reported in grapefruit and other fruits [29]. The ratio of sugar components directly affects sweetness perception. In EY, the ratio of fructose:glucose:sucrose was approximately 1:1:3, whereas it was close to 1:1:1 in EH and EZ, and about 1:1:4 in MD (Table 4). Variations in sugar composition ratios among cultivars may therefore be an important factor leading to differences in perceived sweetness [19]. In stone fruits, TA often exerts a greater influence on the initial sensory impression than sugar content. Citric acid was the predominant organic acid in all cultivars, accounting for 80–90% of total organic acids (Table 5), consistent with previous reports [30]. Malic acid, succinic acid, and oxalic acid were present at lower levels, while tartaric acid was the least abundant and was undetectable in some cultivars. Kang et al. [31] analyzed only citric, malic, and oxalic acids, possibly due to the low contents of succinic and tartaric acids. Malic acid can buffer the sharpness of citric acid, rendering the acidity more mellow and refreshing [32]. Although MD had relatively high TA, its higher malic acid content may soften its overall taste, preventing it from being overly harsh. Significant differences in the contents of the five organic acids among cultivars may be attributed to genetic characteristics and varying growth environments. Compared with Yunnan, green plums grown in Sichuan contained higher levels of malic and succinic acids, which may be a key factor contributing to the clustering differences observed between provinces (Figure 4). A similar trend was reported by Liu et al. [28]. Differences in SSC, total sugar, acidity, and organic acid composition substantially affect fruit flavor, texture, and potential applications, and are influenced by both environmental factors and cultivation practices. Cultivars such as MD and EH, with higher sugar and acidity, are more suitable for high value-added processing, whereas low-sugar and high-acid cultivars such as PX and DY are more suitable for products that require stronger acidity.

Phenolic compounds are important bioactive constituents in green plums, contributing to antioxidant and antimicrobial activities [33]. Gao et al. [34] reported substantial cultivar-dependent variation in TPC among green plums; for instance, Zhaoshui and Yingsu plums exhibited relatively high TPC, whereas Nangao plums contained lower levels. In the present study, DN also showed low TPC, similar to MD (Table 6). Conversely, EY exhibited the highest phenolic content, consistent with the findings of Liu et al. [28], who reported that Yan plum had higher TPC than Zhaoshui and Huang plums. Flavonoids are the main polyphenolic compounds; consequently, cultivars with high TPC often also exhibit elevated flavonoid levels, such as EY and YZ, aligning with the results by Gao et al. [34] However, EZ and EH showed the highest flavonoid contents despite ranking fourth and fifth in TPC, respectively, while PX, with relatively high TPC, contained lower flavonoid levels than MD and DN (Table 6). Thus, the relationship between TPC and flavonoid content is not absolute in all green plums [35], which also partly explains the reason for the low TF/TPC value in eight green plums. Additionally, the VC level of the eight green plums is all lower than that of the Nangao plums grown in Japan [36], but it is similar to that of the green plums from Eastern China [16], which indicated that the composition of the compounds is comprehensively influenced by the local environment, including factors such as sunlight, temperature, and moisture. These three indicators (TPC, TFC, and VC) are commonly used to evaluate the functional potential of green plum cultivars. High phenolic and flavonoid contents in Zhaoshui plum, for example, have been shown to enhance the antioxidant activity of traditional medicinal black plum (Wumei) [34], and phenolics also exhibit antimicrobial and anti-inflammatory effects. In the present study, the antioxidant capacity of the eight cultivars was compared. Interestingly, cultivars could be grouped by geographic origin, with Yunnan-grown plums generally exhibiting higher antioxidant capacity than those from Sichuan (Figure 1), marking a contrast to what was reported by Liu et al. [28]. Within Yunnan, Yan and Zhaoshui plums showed lower antioxidant activity than Sichuan Bai plum and Daqing plum, suggesting that both cultivar and growing location jointly influence antioxidant properties. Across all cultivars, ABTS radical scavenging capacity was higher than DPPH scavenging capacity (Figure 1), which may be attributed to the higher ABTS-scavenging efficiency of chlorogenic acid and VC compared to DPPH [35]. Total phenolics, flavonoids, and VC collectively contribute to the antioxidant capacity of green plums [37]. Therefore, the stronger antioxidant activities of EY and EH may be attributed to their higher levels of these components, whereas PX, MD, and DN showed relatively low TPC and flavonoid contents, and DY exhibited moderate antioxidant capacity due to its relatively higher flavonoid level (Table 6, Figure 1).

Aroma is a critical determinant of fruit quality and consumer acceptance, and it substantially influences the commercial value of fruit products [38]. Different green plums exhibited significant differences in the aroma of fruits and their derived products, with those having richer fruit aroma generally producing more intense fermentation-derived aromas in plum wine. Mogroside V and high hydrostatic pressure were also applied to enrich the aroma of green plum products [39,40]. Ten volatile compounds were important to all cultivars, including ethyl acetate, butyl acetate, and pentyl acetate, which contribute sweet, fruity, banana-like, apple-like, apricot-like, and pineapple-like notes [41,42]. Among them, butyl acetate, hexyl acetate, and butyl butyrate showed relatively high abundances, indicating their importance as characteristic aroma markers. Chen et al. [16] reported that butyl acetate and hexyl acetate are major aroma constituents in Yingsu and Nangao plums, whereas butyl butyrate is more abundant in Baifen plum. In contrast to Liu et al. [15], no terpenoids were detected in any cultivar in the present study, which may be attributed to differences in cultivar and maturity. Chen et al. [16] observed that terpenoid aromas were limited during Changnong plum development and declined to approximately 5% of total volatiles at 70% maturity, with the lowest levels occurring at full ripeness in Nangao and Yingsu plums. Therefore, the absence of terpenoids in this study may be related to the near-mature stage of the sampled fruits. The dominant volatile classes in green plum fruits are influenced by cultivar and maturity [15]. Fully ripe Nangao and Yingsu plums are mainly characterized by esters and alcohols. Consistent with this, DN and DY in this study displayed ester-dominated profiles, followed by alcohols, whereas EY and MD were almost entirely ester-driven (Table 7, Figure 2b). In contrast, YZ, EH, and EY showed pronounced contributions from ketones and aldehydes, aligning with Liu et al.’s classification of seven green plum aroma types. Notably, no cultivar was dominated solely by ketones or aldehydes, and the aroma type of Changnong plum varied markedly across studies [15,16]. EY, EH, and MD exhibited the highest total volatile contents, suggesting their potential as superior raw materials for green plum product development. Additionally, plums grown in Yunnan generally showed higher aroma contents than those from Sichuan, except for EZ. Previous research [43] reported that Nangao plum had the highest aroma among 15 green plum cultivars, including Suiko and Toko, whereas in the present study the volatile content of Nangao plum was relatively low. Therefore, the aroma of green plums is not only influenced by the cultivar but also varies due to regional factors, such as light, temperature, and altitude, and EY, EH, and MD may serve as promising materials for future studies on aroma biosynthesis in green plum.

## 5. Conclusions

This study systematically evaluated the physicochemical properties, antioxidant capacity, and volatile profiles of fruits from eight green plum cultivars. Considerable variation was observed among cultivars in terms of basic physicochemical traits, antioxidant activity, and flavor-related volatile compounds. Cultivars EH and MD exhibited the highest SSC, total sugar, and TA, indicating their potential for high-quality products. In contrast, YZ, EY, EH, and EZ showed high antioxidant activity, particularly in DPPH and ABTS radical scavenging, suggesting their suitability for functional food applications. Volatile profiling revealed 97 major compounds, with esters as the predominant class. Key aroma markers, such as butyl acetate, hexyl acetate, and butyl butyrate, were identified, contributing to the characteristic flavor of green plums. Multivariate analysis identified citric acid as the most influential variable distinguishing cultivars. PCA explained 82.00% of the total variance, and a standardized scoring model classified cultivars into three groups. Class I (MD) was suitable for high-value products like plum wine, while Class II (DY, DN, PX) was better for acid-dominant products, and Class III (EH, EZ, EY, YZ) showed potential for deep processing into functional foods. Overall, these findings provide a theoretical basis for the systematic evaluation of green plum nutritional value and offer guidance for their rational utilization in production and processing. However, this study only comparatively analyzed the influence of cultivars on the quality of green plums. Environmental factors such as light, temperature, and water will be the focus of quality formation in future research.

## Figures and Tables

**Table 1:** Detailed cultivation sites of eight green plum cultivars.

Cultivar	Abbreviated Name	Cultivation Site	Longitude and Latitude
Zhaoshui plum	YZ	Yulong County, Yunnan Province	99°22′ E, 25°53′ N
Yan plum	EY	Eryuan County, Yunnan Province	99°57′ E, 26°07′ N
Huang plum	EH	Eryuan County, Yunnan Province	99°57′ E, 26°07′ N
Zhaoshui plum	EZ	Eryuan County, Yunnan Province	99°57′ E, 26°07′ N
Xing plum	PX	Pingwu County, Sichuan Province	104°33′ E, 32°24′ N
Dabai plum	MD	Mabian County, Sichuan Province	103°26′ E, 28°49′ N
Nangao plum	DN	Dayi County, Sichuan Province	103°32′ E, 30°35′ N
Yingsu plum	DY	Dayi County, Sichuan Province	103°32′ E, 30°35′ N

**Table 2:** Fruit appearance traits of eight green plum cultivars.

Cultivar	Single-Fruit Weight (g)	Transverse Diameter (mm)	Longitudinal Dimension (mm)	Fruit Shape Index
YZ	17.39 ± 0.30 ^b^	30.28 ± 2.42 ^bc^	32.55 ± 0.43 ^abc^	1.07
EY	12.97 ± 0.21 ^c^	32.43 ± 2.04 ^ab^	30.42 ± 1.57 ^cd^	0.94
EH	17.39 ± 1.47 ^b^	30.78 ± 1.46 ^bc^	32.38 ± 0.65 ^abcd^	1.05
EZ	19.16 ± 1.83 ^ab^	35.40 ± 2.11 ^a^	34.45 ± 1.52 ^ab^	0.97
PX	19.78 ± 1.38 ^a^	34.76 ± 1.47 ^a^	35.48 ± 2.04 ^a^	1.02
MD	12.52 ± 1.46 ^c^	26.12 ± 0.04 ^d^	29.41 ± 0.66 ^d^	1.13
DN	12.28 ± 0.37 ^c^	28.75 ± 1.53 ^cd^	31.73 ± 2.10 ^bcd^	1.10
DY	11.48 ± 1.04 ^c^	28.09 ± 1.76 ^cd^	33.03 ± 2.67 ^abc^	1.18

Note: Different letters indicate significant differences among the eight cultivars (*p* < 0.05).

**Table 3:** Soluble solids, total sugars, and total acid content of eight green plum cultivars.

Cultivar	Soluble Solids (°Brix)	Total Sugar (%)	Total Acid (%)	Solid–Acid Ratio	Sugar–Acid Ratio
YZ	8.51 ± 0.02 ^e^	3.19 ± 0.01 ^c^	4.85 ± 0.21 ^e^	1.76 ± 0.08 ^a^	0.66 ± 0.03 ^b^
EY	9.50 ± 0.02 ^c^	2.49 ± 0.03 ^e^	5.37 ± 0.02 ^d^	1.77 ± 0.01 ^a^	0.46 ± 0.01 ^e^
EH	10.10 ± 0.1 ^b^	3.36 ± 0.03 ^b^	6.40 ± 0.06 ^a^	1.58 ± 0.01 ^bc^	0.53 ± 0.01 ^d^
EZ	8.00 ± 0.01 ^f^	3.26 ± 0.01 ^c^	4.95 ± 0.07 ^e^	1.62 ± 0.02 ^b^	0.66 ± 0.02 ^b^
PX	9.05 ± 0.06 ^d^	2.04 ± 0.02 ^f^	5.58 ± 0.09 ^c^	1.62 ± 0.03 ^b^	0.37 ± 0.01 ^f^
MD	10.81 ± 0.02 ^a^	3.70 ± 0.03 ^a^	6.01 ± 0.05 ^b^	1.80 ± 0.02 ^a^	0.62 ± 0.01 ^c^
DN	6.03 ± 0.02 ^g^	2.60 ± 0.14 ^d^	3.41 ± 0.03 ^g^	1.77 ± 0.03 ^a^	0.76 ± 0.05 ^a^
DY	6.10 ± 0.10 ^g^	2.48 ± 0.01 ^e^	3.94 ± 0.11 ^f^	1.55 ± 0.05 ^c^	0.63 ± 0.02 ^bc^

Note: Different letters indicate significant differences among the eight cultivars (*p* < 0.05).

**Table 4:** Fructose, glucose, and sucrose contents of eight green plum cultivars.

Cultivar	Fructose (mg g^−1^)	Glucose (mg g^−1^)	Sucrose (mg g^−1^)	Total Soluble Sugars (mg g^−1^)
YZ	0.92 ± 0.06 ^e^	0.91 ± 0.04 ^e^	0.68 ± 0.02 ^e^	2.51 ± 0.05 ^f^
EY	1.17 ± 0.07 ^c^	1.07 ± 0.02 ^d^	3.15 ± 0.06 ^b^	5.39 ± 0.03 ^c^
EH	2.07 ± 0.06 ^b^	2.32 ± 0.03 ^a^	2.40 ± 0.07 ^c^	6.79 ± 0.10 ^b^
EZ	0.67 ± 0.04 ^f^	0.64 ± 0.03 ^f^	0.80 ± 0.02 ^e^	2.11 ± 0.02 ^g^
PX	0.96 ± 0.01 ^e^	1.41 ± 0.05 ^c^	1.05 ± 0.08 ^d^	3.42 ± 0.08 ^d^
MD	2.17 ± 0.06 ^a^	2.10 ± 0.10 ^b^	7.69 ± 0.16 ^a^	11.96 ± 0.27 ^a^
DN	1.07 ± 0.04 ^d^	1.40 ± 0.05 ^c^	0.25 ± 0.01 ^f^	2.72 ± 0.07 ^e^
DY	0.36 ± 0.03 ^g^	0.36 ± 0.04 ^g^	-	0.72 ± 0.06 ^h^

Note: “-” indicates not detected. Different letters indicate significant differences among the eight cultivars (*p* < 0.05).

**Table 5:** Organic acid contents of eight green plum cultivars.

Cultivar	Organic Acid Content (mg g^−1^ DW)					
	Citric Acid	Malic Acid	Succinic Acid	Oxalic Acid	Tartaric Acid	Total Organic Acid
YZ	462.22 ± 20.41 ^a^	5.67 ± 3.55 ^c^	4.19 ± 0.05 ^g^	3.52 ± 0.00 ^a^	1.40 ± 0.02 ^b^	477.01 ± 23.88 ^ab^
EY	417.52 ± 0.99 ^c^	1.53 ± 0.05 ^c^	14.97 ± 0.01 ^c^	0.98 ± 0.02 ^d^	0.24 ± 0.01 ^d^	435.24 ± 0.94 ^bc^
EH	452.87 ± 14.83 ^ab^	18.81 ± 0.20 ^b^	0.79 ± 0.09 ^h^	0.77 ± 0.02 ^e^	-	473.24 ± 15.11 ^ab^
EZ	275.47 ± 0.51 ^e^	2.12 ± 0.17 ^c^	10.24 ± 0.19 ^f^	0.57 ± 0.00 ^g^	0.50 ± 0.00 ^c^	288.9 ± 0.53 ^e^
PX	326.03 ± 35.50 ^d^	37.46 ± 0.00 ^a^	13.00 ± 0.02 ^d^	0.63 ± 0.00 ^f^	-	377.17 ± 35.44 ^d^
MD	432.29 ± 0.79 ^bc^	44.03 ± 0.76 ^a^	10.78 ± 0.31 ^e^	3.43 ± 0.05 ^b^	1.61 ± 0.07 ^a^	492.14 ± 1.26 ^a^
DN	428.15 ± 13.34 ^bc^	25.81 ± 3.21 ^b^	24.50 ± 0.01 ^a^	3.46 ± 0.03 ^b^	0.18 ± 0.09 ^d^	482.09 ± 16.46 ^a^
DY	346.79 ± 4.58 ^d^	43.87 ± 13.54 ^a^	15.76 ± 0.02 ^b^	1.86 ± 0.04 ^c^	-	408.28 ± 11.33 ^cd^

Note: “-” indicates not detected. Different letters indicate significant differences among the eight cultivars (*p* < 0.05).

**Table 6:** Total phenolic, total flavonoid, and Vitamin C contents of eight green plum cultivars.

Cultivar	Total Phenolic (mg GAE 100 g^−1^ FW)	Total Flavonoids (mg CE 100 g^−1^ FW)	VC (mg 100 g^−1^ FW)
YZ	347.89 ± 3.71 ^b^	11.35 ± 0.80 ^d^	2.00 ± 0.07 ^b^
EY	409.40 ± 12.00 ^a^	16.74 ± 0.20 ^c^	4.30 ± 0.04 ^a^
EH	247.12 ± 12.06 ^d^	19.39 ± 1.41 ^b^	1.13 ± 0.49 ^c^
EZ	296.82 ± 2.52 ^c^	21.13 ± 0.29 ^a^	2.41 ± 0.25 ^b^
PX	359.70 ± 10.71 ^b^	4.26 ± 0.29 ^f^	0.98 ± 0.26 ^c^
MD	82.37 ± 5.96 ^f^	5.57 ± 0.06 ^e^	2.34 ± 0.21 ^b^
DN	91.16 ± 0.82 ^f^	5.22 ± 0.10 ^ef^	2.27 ± 0.25 ^b^
DY	157.61 ± 1.71 ^e^	10.99 ± 0.45 ^d^	0.82 ± 0.05 ^c^

Note: Different letters indicate significant differences among the eight cultivars (*p* < 0.05).

**Table 7:** Volatile components of eight green plum cultivars.

Compound Name	Concentration of Volatile Compounds (μg kg^−1^)							
	YZ	EY	EH	EZ	PX	MD	DN	DY
Number of esters	20	19	15	8	14	11	15	14
Total ester compounds	4265.65 ± 125.94	14,677.96 ± 250.69	8552.44 ± 598.74	563.13 ± 5.39	2481 ± 92.97	6766.12 ± 179.27	1261.15 ± 140.79	3399.77 ± 484.11
Methyl acetate	0 ± 0 ^c^	0 ± 0 ^c^	0 ± 0 ^c^	0 ± 0 ^c^	6.96 ± 0.66 ^b^	0 ± 0 ^c^	0 ± 0 ^c^	27.85 ± 2.16 ^a^
Ethyl acetate	3.85 ± 0.26 ^g^	406.37 ± 7.2 ^d^	53.65 ± 1.31 ^ef^	11.19 ± 0.27 ^fg^	957.84 ± 24.8 ^a^	479.71 ± 13.75 ^c^	63.75 ± 50.43 ^e^	739.38 ± 42.72 ^b^
Butyl acetate	1228.59 ± 6.09 ^d^	7625.3 ± 29.04 ^a^	5459.26 ± 290.46 ^b^	414.27 ± 2.83 ^ef^	742.34 ± 34.34 ^e^	3869.81 ± 94.6 ^c^	337.47 ± 11.32 ^f^	1535.66 ± 407.56 ^d^
Pentyl acetate	48.92 ± 0.88 ^b^	109.87 ± 0.52 ^a^	47.91 ± 31.59 ^b^	6.1 ± 0.18 ^c^	18.2 ± 0.67 ^c^	17.27 ± 1.52 ^c^	17.18 ± 10.14 ^c^	22.00 ± 1.01 ^c^
Hexyl acetate	1263.29 ± 17.69 ^d^	4841.5 ± 193.42 ^a^	2520.34 ± 276.9 ^b^	100.01 ± 0.03 ^f^	564.88 ± 4.24 ^d^	1601.54 ± 50.7 ^c^	463.97 ± 2.16 ^d^	528.91 ± 13.46 ^d^
5-Hexenyl acetate	0 ± 0 ^c^	55.34 ± 1.34 ^bc^	134.08 ± 4.68 ^a^	0 ± 0 ^c^	40.58 ± 0.52 ^bc^	0 ± 0 ^c^	137.13 ± 93.8 ^a^	97.43 ± 2.43 ^ab^
Heptyl acetate	0 ± 0 ^d^	15.67 ± 0.4 ^a^	6.57 ± 0.61 ^b^	0 ± 0 ^d^	0 ± 0 ^d^	0 ± 0 ^d^	3.33 ± 1.9 ^c^	0 ± 0 ^d^
Octyl acetate	0 ± 0 ^c^	39.90 ± 0.3 ^a^	25.63 ± 1.17 ^b^	0 ± 0 ^c^	0 ± 0 ^c^	0 ± 0 ^c^	0 ± 0 ^c^	0 ± 0 ^c^
Benzyl acetate	0 ± 0 ^c^	0 ± 0 ^c^	0 ± 0 ^c^	0 ± 0 ^c^	21.85 ± 10.08 ^a^	0 ± 0 ^c^	2.91 ± 1.24 ^c^	10.12 ± 0.39 ^b^
4-Pentenyl acetate	0 ± 0 ^b^	0 ± 0 ^b^	0 ± 0 ^b^	0 ± 0 ^b^	0 ± 0 ^b^	29.52 ± 8.81 ^a^	0 ± 0 ^b^	0 ± 0 ^b^
Methyl butyrate	5.39 ± 0.15 ^a^	0 ± 0 ^b^	0 ± 0 ^b^	0 ± 0 ^b^	0 ± 0 ^b^	0 ± 0 ^b^	0 ± 0 ^b^	0 ± 0 ^b^
Ethyl butyrate	0 ± 0 ^e^	115.74 ± 3.89 ^b^	0 ± 0 ^e^	2.16 ± 0.28 ^e^	36.45 ± 8.67 ^d^	445.63 ± 2.38 ^a^	7.55 ± 6.43 ^e^	102.62 ± 7.54 ^c^
Butyl butyrate	1218.3 ± 13.81 ^a^	704.86 ± 6.6 ^b^	118.83 ± 9.1 ^cd^	14.62 ± 0.56 ^e^	29.39 ± 0.12 ^e^	79.07 ± 0.77 ^de^	159.5 ± 103.95 ^c^	173.58 ± 2.63 ^c^
Isobutyl butyrate	24.79 ± 0.71 ^a^	0 ± 0 ^b^	0 ± 0 ^b^	0 ± 0 ^b^	0 ± 0 ^b^	0 ± 0 ^b^	0 ± 0 ^b^	0 ± 0 ^b^
Amyl butyrate	103.49 ± 5.64 ^a^	0 ± 0 ^b^	0 ± 0 ^b^	0 ± 0 ^b^	0 ± 0 ^b^	0 ± 0 ^b^	0 ± 0 ^b^	0 ± 0 ^b^
Butyl propionate	60.9 ± 0.86 ^a^	0 ± 0 ^b^	0 ± 0 ^b^	0 ± 0 ^b^	0 ± 0 ^b^	0 ± 0 ^b^	0 ± 0 ^b^	0 ± 0 ^b^
Hexyl propionate	13.38 ± 0.54 ^b^	16.48 ± 0.37 ^a^	0 ± 0 ^d^	0 ± 0 ^d^	0 ± 0 ^d^	0 ± 0 ^d^	7.76 ± 4.83 ^c^	0 ± 0 ^d^
Hexyl isobutyrate	31.61 ± 1.51 ^a^	11.36 ± 0.15 ^b^	0 ± 0 ^c^	0 ± 0 ^c^	0 ± 0 ^c^	0 ± 0 ^c^	0 ± 0 ^c^	0 ± 0 ^c^
Isobutyl isovalerate	17.61 ± 1.63 ^a^	0 ± 0 ^c^	0 ± 0 ^c^	0 ± 0 ^c^	0 ± 0 ^c^	0 ± 0 ^c^	2.62 ± 1.93 ^b^	0 ± 0 ^c^
Hexyl 2-methylbutyrate	71.93 ± 13.38 ^a^	18.87 ± 0.01 ^c^	32.35 ± 3.16 ^b^	0 ± 0 ^d^	0 ± 0 ^d^	0 ± 0 ^d^	8.73 ± 9.12 ^cd^	10.36 ± 0.64 ^cd^
Ethyl hexanoate	21.31 ± 1.91 ^f^	275.41 ± 1.7 ^a^	72.8 ± 1.42 ^d^	0 ± 0 ^g^	34.81 ± 0.33 ^e^	176.48 ± 5.13 ^b^	19.26 ± 5.7 ^f^	100.53 ± 1.97 ^c^
Ethyl octanoate	21.09 ± 0.95 ^b^	155.19 ± 4.73 ^a^	23.67 ± 3.4 ^b^	3.28 ± 0.62 ^c^	11.16 ± 0.77 ^bc^	28.86 ± 0.81 ^b^	24.29 ± 26.93 ^b^	26.48 ± 0.4 ^b^
Octanoic acid, butylester	73.18 ± 38.95 ^b^	218.14 ± 0.21 ^a^	16.58 ± 3.57 ^c^	0 ± 0 ^d^	0 ± 0 ^d^	29.68 ± 0.36 ^cd^	0 ± 0 ^d^	0 ± 0 ^d^
Methyl octanoate	0 ± 0 ^b^	22.55 ± 0.18 ^a^	0 ± 0 ^b^	0 ± 0 ^b^	0 ± 0 ^b^	0 ± 0 ^b^	0 ± 0 ^b^	0 ± 0 ^b^
Hexyl octanoate	6.22 ± 3.32 ^c^	25.49 ± 0.38 ^a^	14.27 ± 0.57 ^b^	0 ± 0 ^d^	0 ± 0 ^d^	0 ± 0 ^d^	0 ± 0 ^d^	0 ± 0 ^d^
Octyl octanoate	0 ± 0 ^b^	14.84 ± 0.14 ^a^	0 ± 0 ^b^	0 ± 0 ^b^	0 ± 0 ^b^	0 ± 0 ^b^	0 ± 0 ^b^	0 ± 0 ^b^
Methyl benzoate	20.18 ± 3.73 ^a^	0 ± 0 ^c^	17.53 ± 0.29 ^b^	0 ± 0 ^c^	0 ± 0 ^c^	0 ± 0 ^c^	0 ± 0 ^c^	0 ± 0 ^c^
Ethyl benzoate	0 ± 0 ^c^	0 ± 0 ^c^	8.97 ± 0.51 ^a^	0 ± 0 ^c^	3.09 ± 0.08 ^b^	0 ± 0 ^c^	0 ± 0 ^c^	0 ± 0 ^c^
Hexyl benzoate	17.01 ± 8.05 ^a^	0 ± 0 ^b^	0 ± 0 ^b^	0 ± 0 ^b^	0 ± 0 ^b^	0 ± 0 ^b^	0 ± 0 ^b^	0 ± 0 ^b^
Ethyl 3-hexenoate	0 ± 0 ^d^	5.08 ± 0.11 ^c^	0 ± 0 ^d^	0 ± 0 ^d^	7.05 ± 2.32 ^b^	0 ± 0 ^d^	0 ± 0 ^d^	21.65 ± 0.14 ^a^
2,2,4-Trimethyl-1,3-pentanediol diisobutyrate	14.61 ± 5.88 ^a^	0 ± 0 ^e^	0 ± 0 ^e^	11.5 ± 0.62 ^ab^	6.4 ± 5.37 ^bcd^	8.55 ± 0.44 ^bc^	5.7 ± 0.64 ^cd^	3.2 ± 1.06 ^de^
Number of aldehydes	5	2	3	3	1	2	4	2
Total aldehyde compounds	102.4 ± 9.6	16.17 ± 0.56	16.85 ± 1.78	11.67 ± 1.83	3.68 ± 1.09	20.35 ± 0.98	17.81 ± 12.89	6.14 ± 0.34
Nonanal	37.71 ± 2.94 ^a^	9.41 ± 0.34 ^bc^	7.54 ± 0.46 ^c^	2.27 ± 0.47 ^de^	0 ± 0 ^e^	11.04 ± 0.62 ^b^	4.31 ± 2.21 ^d^	0 ± 0 ^e^
Decanal	13.79 ± 0.18 ^a^	6.76 ± 0.22 ^c^	4.07 ± 0.02 ^d^	2.17 ± 0.3 ^e^	3.68 ± 1.09 ^d^	9.31 ± 0.36 ^b^	2.24 ± 0.76 ^e^	2.13 ± 0.33 ^e^
2,6-Dimethyl-5-heptenal	1.51 ± 1.02 ^a^	0 ± 0 ^b^	0 ± 0 ^b^	0 ± 0 ^b^	0 ± 0 ^b^	0 ± 0 ^b^	0 ± 0 ^b^	0 ± 0 ^b^
Benzaldehyde	16.82 ± 1.83 ^a^	0 ± 0 ^d^	5.24 ± 1.3 ^bcd^	7.23 ± 1.06 ^bc^	0 ± 0 ^d^	0 ± 0 ^d^	10.82 ± 9.87 ^ab^	4.01 ± 0.01 ^c^
Phenylacetaldehyde	32.57 ± 3.63 ^a^	0 ± 0 ^b^	0 ± 0 ^b^	0 ± 0 ^b^	0 ± 0 ^b^	0 ± 0 ^b^	0 ± 0 ^b^	0 ± 0 ^b^
3,5-Di-tert-butylsalicylaldehyde	0 ± 0 ^b^	0 ± 0 ^b^	0 ± 0 ^b^	0 ± 0 ^b^	0 ± 0 ^b^	0 ± 0 ^b^	0.44 ± 0.05 ^a^	0 ± 0 ^b^
Number of alcohols	8	6	7	8	5	4	9	8
Total alcohol compounds	332.14 ± 18.78	486.58 ± 3.42	545.33 ± 32.17	86.65 ± 2.8	47.16 ± 4.27	99.13 ± 3.84	120.62 ± 63.41	143.17 ± 4.49
1-Butanol	57.17 ± 3.49 ^b^	137.15 ± 0.17 ^a^	98.73 ± 6.66 ^a^	23.35 ± 0.05 ^b^	11.2 ± 0.34 ^b^	50.66 ± 3.07 ^b^	15.18 ± 10.3 ^b^	25.38 ± 0.64 ^b^
2-Methyl-1-butanol	4.2 ± 1.57 ^a^	0 ± 0 ^b^	0 ± 0 ^b^	0 ± 0 ^b^	0 ± 0 ^b^	0 ± 0 ^b^	0 ± 0 ^b^	0 ± 0 ^b^
Hexyl alcohol	181.54 ± 6.46 ^c^	252.42 ± 1.63 ^b^	337.49 ± 13.27 ^a^	35.34 ± 0.73 ^e^	22.18 ± 0.74 ^e^	37.82 ± 0.38 ^e^	71.12 ± 42.49 ^d^	43.1 ± 0.08 ^e^
3-Hexen-1-ol	9.35 ± 1.44 ^a^	0 ± 0 ^b^	0 ± 0 ^b^	0 ± 0 ^b^	0 ± 0 ^b^	0 ± 0 ^b^	0 ± 0 ^b^	0 ± 0 ^b^
Trans-2-Hexen-1-ol	21.95 ± 1.3 ^a^	0 ± 0 ^b^	0 ± 0 ^b^	0 ± 0 ^b^	0 ± 0 ^b^	0 ± 0 ^b^	0 ± 0 ^b^	0 ± 0 ^b^
Trans-3-Hexen-1-ol	0 ± 0 ^b^	9.48 ± 0.13 ^a^	0 ± 0 ^b^	0 ± 0 ^b^	0 ± 0 ^b^	0 ± 0 ^b^	0 ± 0 ^b^	0 ± 0 ^b^
4-Methyl-1-hexanol	4.43 ± 0.99 ^ab^	0 ± 0 ^c^	0 ± 0 ^c^	0 ± 0 ^c^	4.98 ± 0.32 ^a^	4.5 ± 0.18 ^ab^	0 ± 0 ^c^	4.04 ± 0.05 ^b^
5-Hexen-1-ol	0 ± 0 ^b^	0 ± 0 ^b^	0 ± 0 ^b^	0 ± 0 ^b^	0 ± 0 ^b^	0 ± 0 ^b^	7.3 ± 2.96 ^a^	0 ± 0 ^b^
Linalool	19.76 ± 2.34 ^c^	46.39 ± 1.24 ^b^	53.04 ± 2.12 ^a^	12.71 ± 0.44 ^de^	0 ± 0 ^f^	0 ± 0 ^f^	10.38 ± 2.66 ^e^	13.65 ± 0.61 ^d^
Alpha-Terpineol	0 ± 0 ^c^	18.28 ± 0.14 ^a^	8.9 ± 3.87 ^b^	0 ± 0 ^c^	0 ± 0 ^c^	0 ± 0 ^c^	2.57 ± 0.63 ^c^	0 ± 0 ^c^
α-Terpineol	0 ± 0 ^c^	0 ± 0 ^c^	0 ± 0 ^c^	3.45 ± 0.39 ^a^	0 ± 0 ^c^	0 ± 0 ^c^	2.42 ± 0.71 ^b^	0 ± 0 ^c^
4-(2,6,6-trimethyl-2-cyclohexen-1-yl)-3-Buten-a-2-ol	0 ± 0 ^b^	0 ± 0 ^b^	3.82 ± 2.36 ^a^	0 ± 0 ^b^	0 ± 0 ^b^	0 ± 0 ^b^	0 ± 0 ^b^	0 ± 0 ^b^
Dihydro-beta-ionol	33.74 ± 18.19 ^a^	22.86 ± 0.11 ^a^	34.73 ± 3.72 ^a^	3.69 ± 0.69 ^b^	0 ± 0 ^b^	6.15 ± 0.21 ^b^	3.42 ± 1.25 ^b^	0 ± 0 ^b^
Heptyl alcohol	0 ± 0 ^c^	0 ± 0 ^c^	0 ± 0 ^c^	5.12 ± 0.3 ^ab^	5.43 ± 0.52 ^a^	0 ± 0 ^c^	4.69 ± 0.66 ^b^	0 ± 0 ^c^
2-Ethylhexanol	0 ± 0 ^c^	0 ± 0 ^c^	0 ± 0 ^c^	2.04 ± 0.07 ^b^	0 ± 0 ^c^	0 ± 0 ^c^	0 ± 0 ^c^	20.82 ± 0.69 ^a^
1-Phenyl-2-propanol	0 ± 0 ^b^	0 ± 0 ^b^	0 ± 0 ^b^	0.95 ± 0.13 ^a^	0 ± 0 ^b^	0 ± 0 ^b^	0 ± 0 ^b^	0 ± 0 ^b^
Benzyl alcohol	0 ± 0 ^b^	0 ± 0 ^b^	0 ± 0 ^b^	0 ± 0 ^b^	3.37 ± 2.35 ^a^	0 ± 0 ^b^	0 ± 0 ^b^	0 ± 0 ^b^
6-Methyl-5-hepten-2-ol	0 ± 0 ^b^	0 ± 0 ^b^	8.62 ± 0.17 ^a^	0 ± 0 ^b^	0 ± 0 ^b^	0 ± 0 ^b^	0 ± 0 ^b^	0 ± 0 ^b^
3-Methyl-2-butanol	0 ± 0 ^b^	0 ± 0 ^b^	0 ± 0 ^b^	0 ± 0 ^b^	0 ± 0 ^b^	0 ± 0 ^b^	0 ± 0 ^b^	6.05 ± 0.79 ^a^
(R)-(-)-2-Pentanol	0 ± 0 ^c^	0 ± 0 ^c^	0 ± 0 ^c^	0 ± 0 ^c^	0 ± 0 ^c^	0 ± 0 ^c^	3.54 ± 1.75 ^b^	27.87 ± 1.18 ^a^
2,6-Dimethyl-3,7-octadiene-2,6-diol	0 ± 0 ^b^	0 ± 0 ^b^	0 ± 0 ^b^	0 ± 0 ^b^	0 ± 0 ^b^	0 ± 0 ^b^	0 ± 0 ^b^	2.26 ± 0.45 ^a^
Number of ketones	7	5	6	6	4	1	3	2
Total ketone compounds	932.63 ± 37.96	471.87 ± 5.79	2634.44 ± 55.66	121.67 ± 1.21	47.35 ± 4.48	75.76 ± 2.35	134.11 ± 64.27	78.22 ± 4.11
2-Octanone	85.93 ± 7.73 ^b^	48.01 ± 0.72 ^bc^	60.94 ± 6.75 ^bc^	70.13 ± 0.22 ^b^	25.71 ± 2.32 ^c^	75.76 ± 2.35 ^b^	123.98 ± 59.38 ^a^	73.7 ± 3.21 ^b^
6-Methyl-5-hepten-2-one	63.05 ± 8.23 ^a^	0 ± 0 ^b^	1.5 ± 0.21 ^b^	0 ± 0 ^b^	0 ± 0 ^b^	0 ± 0 ^b^	0 ± 0 ^b^	0 ± 0 ^b^
1-p-Tolyl-pentan-1-one	13.9 ± 0.86 ^a^	0 ± 0 ^d^	0 ± 0 ^d^	7.22 ± 0.19 ^b^	0 ± 0 ^d^	0 ± 0 ^d^	5.18 ± 0.32 ^c^	0 ± 0 ^d^
3′-Hydroxyacetophenone	1.18 ± 0.16 ^a^	0 ± 0 ^b^	0 ± 0 ^b^	0 ± 0 ^b^	0 ± 0 ^b^	0 ± 0 ^b^	0 ± 0 ^b^	0 ± 0 ^b^
Dihydro-beta-ionone	412.65 ± 11 ^a^	162.59 ± 0.23 ^c^	213.43 ± 22.06 ^b^	42.27 ± 0.46 ^d^	2.46 ± 1.97 ^e^	0 ± 0 ^e^	4.95 ± 4.57 ^e^	4.52 ± 0.9 ^e^
Geranylacetone	21.72 ± 3.86 ^b^	25.66 ± 0.22 ^a^	0 ± 0 ^d^	0 ± 0 ^d^	3.05 ± 0.06 ^c^	0 ± 0 ^d^	0 ± 0 ^d^	0 ± 0 ^d^
4-(2,6,6-Trimethyl-1-cyclohexenyl)-3-buten-2-one	334.2 ± 86.12 ^a^	0 ± 0 ^b^	0 ± 0 ^b^	0 ± 0 ^b^	16.13 ± 0.13 ^b^	0 ± 0 ^b^	0 ± 0 ^b^	0 ± 0 ^b^
β-Ionone	0 ± 0 ^c^	224.13 ± 4.25 ^b^	630.89 ± 14.59 ^a^	0 ± 0 ^c^	0 ± 0 ^c^	0 ± 0 ^c^	0 ± 0 ^c^	0 ± 0 ^c^
Alpha-Ionone	0 ± 0 ^c^	11.48 ± 0.37 ^b^	17.8 ± 2.87 ^a^	0 ± 0 ^c^	0 ± 0 ^c^	0 ± 0 ^c^	0 ± 0 ^c^	0 ± 0 ^c^
Acetophenone	0 ± 0 ^b^	0 ± 0 ^b^	0 ± 0 ^b^	2.05 ± 0.34 ^a^	0 ± 0 ^b^	0 ± 0 ^b^	0 ± 0 ^b^	0 ± 0 ^b^
Acetoin	0 ± 0 ^b^	0 ± 0 ^b^	1709.88 ± 9.18 ^a^	0 ± 0 ^b^	0 ± 0 ^b^	0 ± 0 ^b^	0 ± 0 ^b^	0 ± 0 ^b^
Number of acids	5	0	0	3	4	3	2	4
Total acid compounds	62.84 ± 17.85	0 ± 0	0 ± 0	16.15 ± 0.99	36.01 ± 2.14	32.16 ± 2.2	24.64 ± 17.34	30.01 ± 1.23
Acetic acid	0 ± 0 ^d^	0 ± 0 ^d^	0 ± 0 ^d^	0 ± 0 ^d^	7.52 ± 0.5 ^c^	23.92 ± 0.72 ^a^	0 ± 0 ^d^	12.51 ± 0.52 ^b^
Butyric acid	7.84 ± 1.91 ^a^	0 ± 0 ^b^	0 ± 0 ^b^	0 ± 0 ^b^	0 ± 0 ^b^	0 ± 0 ^b^	0 ± 0 ^b^	0 ± 0 ^b^
Valeric acid	0 ± 0 ^d^	0 ± 0 ^d^	0 ± 0 ^d^	8.55 ± 0.47 ^a^	1.45 ± 0.51 ^cd^	5.37 ± 1.12 ^b^	2.45 ± 2.35 ^c^	2.31 ± 0.2 ^c^
Hexanoic acid	39.84 ± 9.75 ^a^	0 ± 0 ^c^	0 ± 0 ^c^	6.22 ± 0.38 ^c^	21.37 ± 0.55 ^b^	0 ± 0 ^c^	22.19 ± 14.99 ^b^	0 ± 0 ^c^
Octanoic acid	3.37 ± 0.96 ^a^	0 ± 0 ^b^	0 ± 0 ^b^	0 ± 0 ^b^	0 ± 0 ^b^	0 ± 0 ^b^	0 ± 0 ^b^	0 ± 0 ^b^
Nonanoic acid	2.37 ± 0.77 ^b^	0 ± 0 ^d^	0 ± 0 ^d^	1.38 ± 0.14 ^c^	0 ± 0 ^d^	2.87 ± 0.36 ^b^	0 ± 0 ^d^	14.53 ± 0.46 ^a^
Pentadecanoic acid	9.42 ± 4.46 ^a^	0 ± 0 ^b^	0 ± 0 ^b^	0 ± 0 ^b^	0 ± 0 ^b^	0 ± 0 ^b^	0 ± 0 ^b^	0 ± 0 ^b^
Trans-3-Hexenoic acid	0 ± 0 ^c^	0 ± 0 ^c^	0 ± 0 ^c^	0 ± 0 ^c^	5.67 ± 0.58 ^a^	0 ± 0 ^c^	0 ± 0 ^c^	0.66 ± 0.05 ^b^
Number of other compounds	15	5	2	2	5	0	6	6
Total of other compounds	697.6 ± 66.52	164.07 ± 1.86	134.4 ± 21.56	29.4 ± 0.58	201.24 ± 12.76	0 ± 0	285.89 ± 90.28	214.98 ± 16.58
1,2-Xylene	110.53 ± 0.5 ^a^	0 ± 0 ^b^	0 ± 0 ^b^	0 ± 0 ^b^	0 ± 0 ^b^	0 ± 0 ^b^	0 ± 0 ^b^	0 ± 0 ^b^
1,3-Xylene	42.28 ± 0.3 ^a^	0 ± 0 ^b^	0 ± 0 ^b^	0 ± 0 ^b^	0 ± 0 ^b^	0 ± 0 ^b^	0 ± 0 ^b^	0 ± 0 ^b^
(+)-Limonene	70.31 ± 49 ^a^	0 ± 0 ^b^	0 ± 0 ^b^	0 ± 0 ^b^	0 ± 0 ^b^	0 ± 0 ^b^	14.37 ± 5.52 ^b^	0 ± 0 ^b^
Styrene	151.97 ± 1.54 ^a^	14.22 ± 0.58 ^d^	122.67 ± 21.55 ^b^	25.33 ± 0.57 ^cd^	21.11 ± 0.19 ^cd^	0 ± 0 ^d^	44.56 ± 39.98 ^c^	47.58 ± 1.14 ^c^
1,2,3-Trimethylbenzene	16.68 ± 0.59 ^a^	0 ± 0 ^b^	0 ± 0 ^b^	0 ± 0 ^b^	0 ± 0 ^b^	0 ± 0 ^b^	0 ± 0 ^b^	0 ± 0 ^b^
M-Cymene	18.49 ± 3.32 ^a^	0 ± 0 ^b^	0 ± 0 ^b^	0 ± 0 ^b^	0 ± 0 ^b^	0 ± 0 ^b^	0 ± 0 ^b^	0 ± 0 ^b^
Theaspirane	34.84 ± 16.07 ^a^	19.23 ± 0.23 ^b^	11.73 ± 0.01 ^b^	0 ± 0 ^c^	0 ± 0 ^c^	0 ± 0 ^c^	0 ± 0 ^c^	0 ± 0 ^c^
2-Nitropropane	41.54 ± 1.88 ^a^	0 ± 0 ^b^	0 ± 0 ^b^	0 ± 0 ^b^	0 ± 0 ^b^	0 ± 0 ^b^	0 ± 0 ^b^	0 ± 0 ^b^
Ethylbenzene	30.2 ± 33.36 ^a^	0 ± 0 ^b^	0 ± 0 ^b^	0 ± 0 ^b^	0 ± 0 ^b^	0 ± 0 ^b^	0 ± 0 ^b^	0 ± 0 ^b^
Tridecane	9.35 ± 0.4 ^a^	0 ± 0 ^b^	0 ± 0 ^b^	0 ± 0 ^b^	0 ± 0 ^b^	0 ± 0 ^b^	0 ± 0 ^b^	0 ± 0 ^b^
1,4-Dichlorobenzene	10.13 ± 0.6 ^a^	0 ± 0 ^b^	0 ± 0 ^b^	0 ± 0 ^b^	0 ± 0 ^b^	0 ± 0 ^b^	0 ± 0 ^b^	0 ± 0 ^b^
4-Hexanolide	12.97 ± 2.36 ^b^	19.8 ± 0.12 ^a^	0 ± 0 ^d^	0 ± 0 ^d^	7.48 ± 2.22 ^c^	0 ± 0 ^d^	6.89 ± 2.95 ^c^	9.2 ± 1 ^c^
4-Hydroxybutyric acid gamma-la	0 ± 0 ^b^	0 ± 0 ^b^	0 ± 0 ^b^	0 ± 0 ^b^	0 ± 0 ^b^	0 ± 0 ^b^	0 ± 0 ^b^	64.37 ± 2.27 ^a^
R-γ-Decalactone	103.42 ± 21.48 ^a^	91.15 ± 0.83 ^ab^	0 ± 0 ^d^	0 ± 0 ^d^	76.87 ± 3.57 ^b^	0 ± 0 ^d^	40.54 ± 16.39 ^c^	14.86 ± 0.61 ^d^
gamma-Octanoic lactone	0 ± 0 ^b^	19.67 ± 0.1 ^a^	0 ± 0 ^b^	0 ± 0 ^b^	0 ± 0 ^b^	0 ± 0 ^b^	0 ± 0 ^b^	0 ± 0 ^b^
2,4-Di-tert-butylphenol	43.03 ± 2.59 ^d^	0 ± 0 ^e^	0 ± 0 ^e^	0 ± 0 ^e^	95.6 ± 6.6 ^b^	0 ± 0 ^e^	178.11 ± 24.92 ^a^	75.16 ± 2.11 ^c^
2-Valerylfuran	0 ± 0 ^b^	0 ± 0 ^b^	0 ± 0 ^b^	0 ± 0 ^b^	0.18 ± 0.18 ^a^	0 ± 0 ^b^	0 ± 0 ^b^	0 ± 0 ^b^
1-Methyl-4-methylenecyclohexane.	0 ± 0 ^b^	0 ± 0 ^b^	0 ± 0 ^b^	4.07 ± 0.01 ^a^	0 ± 0 ^b^	0 ± 0 ^b^	0 ± 0 ^b^	0 ± 0 ^b^
3,3-Dimethylhexane	0 ± 0 ^b^	0 ± 0 ^b^	0 ± 0 ^b^	0 ± 0 ^b^	0 ± 0 ^b^	0 ± 0 ^b^	1.42 ± 0.52 ^a^	0 ± 0 ^b^
1-Pentene,4,4-dimethyl	1.86 ± 0.53 ^b^	0 ± 0 ^c^	0 ± 0 ^c^	0 ± 0 ^c^	0 ± 0 ^c^	0 ± 0 ^c^	0 ± 0 ^c^	3.81 ± 0.45 ^a^

Note: Different letters indicate significant differences among the eight cultivars (*p* < 0.05).

**Table 8:** Principal component scores and comprehensive quality scores of eight green plum cultivars.

Cultivar	Principal Component Score			Y	Rank
	Y_1_	Y_2_	Y_3_		
EH	2.979	1.106	− 0.427	1.515	1
MD	− 0.062	4.056	− 0.273	1.206	2
EY	1.677	− 1.161	0.668	0.392	3
YZ	1.537	− 0.961	− 0.650	0.262	4
PX	− 1.387	0.289	2.452	− 0.226	5
EZ	0.971	− 1.999	− 0.257	− 0.250	6
DY	− 2.149	− 1.427	− 0.430	− 1.363	7
DN	− 3.566	0.097	− 1.082	− 1.534	8

## Data Availability

The original contributions presented in this study are included in the article/Supplementary Materials. Further inquiries can be directed to the corresponding author.

## Supplementary Materials

The following supporting information can be downloaded at https://www.mdpi.com/article/10.3390/foods15061057/s1, Table S1: Eigenvalues, contribution rates and cumulative contribution rates of principal components. Table S2: Factor loading matrix of principal components on each quality index. Table S3: Analytical validation parameters for quantitative methods by HPLC.
