# Supplementary material for: Comparative Analysis of Physicochemical Properties and Volatile Profile of Eight Varieties of Green Plums in Sichuan and Yunnan
Source: Foods. 2026 Mar 17;15(6):1057. doi: 10.3390/foods15061057 (PMC13025654; doi:10.3390/foods15061057)
Supplement: Supplementary file 1 [file foods-15-01057-s001.zip › foods-4171951-supplementary.pdf]

Caption:

Table S1 Eigenvalues, contribution rates and cumulative contribution rates of principal components.

Table S2 Factor loading matrix of principal components on each quality index.

Table S3 Analytical validation parameters for quantitative methods by HPLC

Table S1 Eigenvalues, contribution rates and cumulative contribution rates of principal components

**Principal Components**	**Eigenvalue**	**Variance contribution rate (%)**	**Cumulative variance contribution rate (%)**
1	4.893	40.777	40.777
2	3.724	31.037	71.814
3	1.223	10.189	82.003

Table S2 Factor loading matrix of principal components on each quality index

**Indicators**	**Number**	**Principal Components**		
		**1**	**2**	**3**
Total sugar	X1	0.525	0.465	-0.588
Total acid	X2	0.743	0.514	0.396
Total phenolic	X3	0.553	-0.535	0.571
Soluble solids	X4	0.704	0.607	0.336
DPPH	X5	0.849	-0.434	-0.049
ABTS	X6	0.823	-0.492	-0.183
Citric acid	X7	0.263	0.454	-0.414
Succinic acid	X8	-0.852	-0.139	-0.001
Malic acid	X9	-0.611	0.565	0.155
Sucrose	X10	0.291	0.825	0.032
Flavonoids	X11	0.732	-0.473	-0.221
Glucose	X12	0.274	0.842	0.035

Table S3 Analytical validation parameters for quantitative methods by HPLC

Parameter category	Analyte	Regression Equation	R^2	Linear Range	LOD (mg/L)	LOQ (mg/L)	Precision RSD (standard substance)	Repeatability RSD (green plum)	Stability RSD (green plum)	Recovery (green plum)	Recovery RSD
soluble sugars	Fructose	y = 93.82x - 172.7047	0.9999	10.018-400.741μg·mL^{-1}	4.61	13.96	0.18% (0.1mg·mL^{-1}）	3.95% (YM)	0.13% (YZ)	94.58% (YM)	2.62% (YM)
	Glucose	y=41.17x+1.8245	0.9995	9.977-399.091 μg·mL^{-1}	12.46	37.77	0.14% (0.1mg·mL^{-1}）	3.04% (YM)	0.24% (YZ)	96.83% (YM)	1.73% (YM)
	Sucrose	y = 99.33x - 50.5549	0.9998	10.018-400.731μg·mL^{-1}	8.65	26.21	0.17% (0.1mg·mL^{-1}）	1.77% (YM)	0.18% (YZ)	97.20% (YM)	1.26% (YM)
organic acids	Citric acid	y = 1140.30x - 1.4085	0.9999	20.068-1003.42μg·mL^{-1}	16.23	49.19	0.45% (0.2mg·mL^{-1}）	3.08% (YZ)	0.23% (YZ)	97.23% (YZ)	1.54% (YZ)
	Malic acid	y = 850.70x + 2.6507	0.9998	20.286-1014.33μg·mL^{-1}	15.53	47.06	0.46% (0.2mg·mL^{-1}）	2.75% (YZ)	1.36% (YZ)	94.92% (YZ)	2.17% (YZ)
	Succinic acid	y = 549.39x - 1.2587	0.9999	9.289-464.446 μg·mL^{-1}	7.98	24.17	1.53% (0.1mg·mL^{-1}）	0.96% (YZ)	1.84% (YZ)	96.12% (YZ)	2.14% (YZ)
	Oxalic acid	y=10263.01x - 5.1864	0.9997	2.531-126.532 μg·mL^{-1}	4.3	13.02	0.53% (0.025mg·mL^{-1}）	2.57% (YZ)	0.31% (YZ)	94.82% (YZ)	2.33% (YZ)
	Tartaric acid	y = 1864.86x + 6.6448	0.9999	20.284-1014.18μg·mL^{-1}	19.37	58.69	1.08% (0.1mg·mL^{-1}）	1.76% (YZ)	2.24% (YZ)	97.10% (YZ)	1.66% (YZ)
